# Forage grass growth under future climate change scenarios affects fermentation and ruminant efficiency

**DOI:** 10.1038/s41598-022-08309-7

**Published:** 2022-03-15

**Authors:** Elizabeth H. Hart, Sarah R. Christofides, Teri E. Davies, Pauline Rees Stevens, Christopher J. Creevey, Carsten T. Müller, Hilary J. Rogers, Alison H. Kingston-Smith

**Affiliations:** 1 Institute of Biological, Environmental and Rural Sciences (IBERS), Aberystwyth University, Aberystwyth, Wales SY23 3FG UK; 2 School of Biosciences, Cardiff University, The Sir Martin Evans Building, Museum Avenue, Cardiff, CF10 3AX UK; 3 School of Biological Sciences, Queens University Belfast, Belfast, BT9 7BL UK

**Keywords:** Plant stress responses, Applied microbiology

## Abstract

With an increasing human population access to ruminant products is an important factor in global food supply. While ruminants contribute to climate change, climate change could also affect ruminant production. Here we investigated how the plant response to climate change affects forage quality and subsequent rumen fermentation. Models of near future climate change (2050) predict increases in temperature, CO_2_, precipitation and altered weather systems which will produce stress responses in field crops. We hypothesised that pre-exposure to altered climate conditions causes compositional changes and also primes plant cells such that their post-ingestion metabolic response to the rumen is altered. This “stress memory” effect was investigated by screening ten forage grass varieties in five differing climate scenarios, including current climate (2020), future climate (2050), or future climate plus flooding, drought or heat shock. While varietal differences in fermentation were detected in terms of gas production, there was little effect of elevated temperature or CO_2_ compared with controls (2020). All varieties consistently showed decreased digestibility linked to decreased methane production as a result of drought or an acute flood treatment. These results indicate that efforts to breed future forage varieties should target tolerance of acute stress rather than long term climate.

## Introduction

Forage crops for livestock are essential for ruminant production, with grazing land accounting for approximately 60% of global agriculture land^1^. Continued human population growth predicts an increase to 9.7 billion by 2050, which will lead to an increase in demand for animal products together with pressure to decrease pollutant output. Increases in ruminant production have been achieved to date through continual improvement of the forage feed germplasm focusing mainly on traits such as yield and digestibility^2^. It can take over 10 years from breeding to release of a new variety and so current varieties are tailored to perform well under current environmental conditions. However, it is recognised that climate is changing; climate change models of the near future (2050) predict increases in temperature of 4–6 °C in the UK, atmospheric CO_2_ from 400 to 500 ppm^3^, increases in precipitation (up to 33% more), and altered weather systems (e.g. extreme drought and flooding^4^). The forage varieties currently used in UK are adapted to current conditions. However, growth under elevated temperature, drought or flood can induce stress responses in the grass^5^ that could affect not just production but also composition, and thereby nutritive value to ruminants; for instance forage quality has been shown to declines with rising temperatures^6^. Therefore, to secure future productivity of livestock the development of new forage varieties should take account of the environmental effects on grass production and quality parameters.

Ruminants have the ability to convert fibrous feed unable to be utilised by humans, into easily digestible meat and milk products^7,8^ due to the presence of a complex rumen microbiome^9^. However, perturbation of the rumen can be brought about by diet and diet based effects have been observed on both the core microbial community^10^ and overall rumen microbial community^11–14^. Previous research has indicated improvement of rumen efficiency could be achieved by manipulating animal diet, improving host-microbial interactions and plant microbial interactions to maximise productivity whilst reducing environmental costs^15^.

Ingested conserved forages are broken down in the rumen by the action of enzymes from the attached rumen microbiota, but when fresh forage is ingested in addition to microbial activity the possibility exists for a contribution to feed degradation from plant metabolism caused by initiation of innate plant stress responses^16–19^. When fresh forage is fed to ruminants the cells are still metabolically active, and respond to the conditions of the rumen, including by inducing plant hormone-mediated stress responses resulting in autolytic breakdown^20^. These post-ingestion changes in plant-based metabolism have the potential to alter substrate composition and as a consequence could affect fermentation profiles in forages with apparently similar chemical composition^2^. It is currently unclear what the relative contributions of plant and microbial proteolysis to overall rumen function are. However, the possibility that there is a contribution from the plant cells offers an exciting opportunity to exploit natural variation in endogenous plant proteolysis. Selecting for grass varieties that have slow rates of endogenous proteolysis might at least partially mitigate against environmental pollution^21,22^. However, evidence is limited on the effect of climate change scenarios on the fermentation characteristics of plants which have been challenged by these stresses.

Plants respond to abiotic and biotic stresses by physiological, biochemical, metabolic and molecular mechanisms^23^. Previous studies have focussed on the characterisation of physiological changes of forage plant varieties under climate change conditions. For example, elevated levels of CO_2_ have been shown to improve the rate of photosynthesis in plants^24^ and alleviate the effects of drought stress by conserving water, increasing carbon fixation and increasing fructan accumulation^25^. However high temperatures have been shown to inhibit photosynthesis^26^ by altering the structure of chloroplasts and by inactivating chloroplast enzymes through oxidative stress^27^. Drought stress has also been shown to affect water soluble carbohydrate (WSC) levels. Concentration of fructans in forage grasses had a higher degree of polymerisation in drought than in control conditions, although the increase in WSC reserves to the level of 40 to 50% at the end of drought were not thought to improve drought tolerance in the plants overall^28^. Previous studies have also demonstrated that drought can decrease the protein concentration due to an increase in protein degradation, a decrease in nitrogen assimilation and a decrease in protein synthesis^29^. During flooding oxidative pathways within the plant move towards fermentation^30^ and CO_2_ levels within plants can become reduced. This leads to a decreased electron sink for light energy and, potentially, oxidative damage of plant cells^31^ from excess light energy not used in CO_2_ fixation^32^. In flooding scenarios where both root and shoot are submerged, aerobic respiration and photosynthesis is reduced due to low light levels and limited gaseous exchange leading to an energy crisis within the plant^33^.

Plants have the ability to “remember” past occurrences to adapt to new environments^34^. Pre-exposure to environmental stresses primes the plant cells such that the subsequent response is altered^35^. This stress memory is now understood to be a key factor determining progression of microbial disease in crops mediated by salicylic acid^36^ and abiotic responses to temperature^37^. Salicylic acid has been implicated in the response of plant cells to the rumen^20^, and thus, pre-exposure of forage plants to stress during growth may prime the cells, such that the stress response is altered once ingested and in the rumen^19^. Hence an active response may have important effects on rumen fermentation efficiency and subsequently host nutrient uptake in addition to direct effects of climate on the chemical composition of the forage.

Despite extensive study of responses of crop and forage species to adverse environmental conditions, there has been relatively little research into how forage adaptation to climate change will affect ruminant production systems, and the appropriateness of current and future forage varieties for animal production in the future. The aim of this work was therefore to determine how responses to the growth environment, including “stress memory” may affect the subsequent forage degradation in the rumen and, therefore, to understand how ruminant feeding strategies might be affected by climate change.

## Materials and methods

### Plant material

Ten different current commercial varieties of forage grasses (Table 1) were grown for 3 months in five different climate scenarios, replicated three times at 1-week intervals. Studies on these plant materials were undertaken in compliance with local and national regulations. Climate conditions were simulated in growth chambers and consisted of present-day conditions and future potential climate scenarios as described by Ref.^38^ (Table 2).

**Table 1:** Forage grass varieties.

Forage grass	Type
AberClyde	Tetraploid perennial ryegrass, high sugar (*Lolium perenne*)
AberDart	Diploid perennial ryegrass, high sugar (*Lolium perenne*)
AberEcho	Tetraploid hybrid ryegrass, high sugar (*Lolium x boucheanum*)
AberGlyn	Tetraploid perennial ryegrass (*Lolium perenne*)
AberNiche	Tetraploid festulolium (Italian ryegrass × meadow fescue) (*Lolium multiflorum × Festuca pratense*)
AberRoot	Perennial tetraploid festulolium, high sugar (perennial ryegrass × Atlas fescue) (*Lolium perenne × Festuca mairei*)
AberZeus	Diploid perennial ryegrass, high sugar (*Lolium perenne*)
Barolex	Allohexaploid, tall fescue *(Festuca arundinacea*)
Davinci	Diploid Italian ryegrass (*Lolium multiflorum*)
Premium	Diploid perennial ryegrass (*Lolium perenne*)

**Table 2:** Climate conditions.

	Climate condition	Description
1	Control 2020	400 ppm CO_2,_ 16–18 °C night/day temperature for an 8-h photoperiod and watered regularly
2	Control 2050	500 ppm CO_2,_ 21–23 °C night/day temperature for an 8-h photoperiod and watered regularly
3	Flood	As for control 2050 but flooded for 1 week prior to harvesting
4	Drought	As for control 2050 but no water for 1 week prior to harvesting
5	Heat Shock	As for control 2050 but temperature increased to 35 °C 2 days prior to harvesting

### Forage characterisation

For each climate scenario, after the 3-month growth period and before harvest, leaves of each variety were analysed for Fv/Fm (Handy PEA, Hansatech Instruments, UK) to determine photochemical efficiency of each plant in each environment^39^. Chlorophyll content was determined by the addition of 80% acetone to 1 g of fresh plant tissue, which was placed in the dark for 1 h. The sample was then centrifuged for 5 min at 15,000×*g* and the absorbance at λ = 663 nm (A_663_) and 645 nm (A_645_) of the supernatant was determined spectrophotometrically (Pharmacia Biotech). Chlorophyll content was calculated from absorbencies according to Ref.^40^ and also used as proxy for stress response. Plant dry matter and crude protein were determined according to methods 930.15 and 968.06 respectively (AOAC^41^), and water-soluble carbohydrates (WSC) spectrophotometrically using the anthrone method^42^. Dried plant material was analysed sequentially for neutral detergent fibre (NDF), acid detergent fibre (ADF) and acid detergent lignin (ADL)^43,44^ by using a fibre analyser (ANKOM). Leaf samples of each grass were also freeze dried and ground to a fine powder in a ball mill (MM 30, Retsch Gmbh, Haan,Germany) at speed 30 for 2 min with the inclusion of 2 tungsten beads previously washed in acetone. Ground leaf material was subjected to Fourier transform infrared spectroscopy (FTIR) (Equinox 55 HTS-XT FTIR Spectrophotometer, Bruker UK Ltd, Coventry, UK). This enabled non-targeted analysis (profiling) of biochemical changes within the plant tissue during growth conditions.

### In vitro batch fermentation

#### Collection and preparation of microbial inoculum

All experiments involving animals were performed in accordance with relevant guidelines and regulations. Experimentation was conducted under the authority of licenses under the U.K. Animals (Scientific Procedures) Act, 1986. Experimentation was approved by the Aberystwyth University Animal Welfare and Ethical Review Body (AWERB). Methods are reported here in accordance with Animal Research: Reporting of In Vivo Experiments (ARRIVE) guidelines (https://arriveguidelines.org). Rumen fluid was collected on the morning of each plant harvest from each of four non-lactating Holstein–Friesian dairy cows which had been previously prepared with rumen cannulae (Bar Diamond, Parma, ID), and fed *ad-lib* perennial ryegrass (*L. perenne*). The rumen samples were mixed and filtered through two layers of muslin under a CO_2_ stream and the filtrate used to prepare a 10% rumen fluid inoculum in anaerobic buffer^44^.

#### Analysis of gas production

Each gas production run consisted of triplicate samples from each of 50 treatments with the addition of inoculum blanks and was repeated over three consecutive weeks. On the day prior to harvest a subsample of each plant was dried to a constant weight to determine DM. Fresh leaves from grass varieties grown in each climate scenario (n = 50) were cut into approx. 1 cm lengths and accurately weighed in triplicate into 140 mL serum bottles to supply fresh matter equivalent to 1 g DM, which were then filled with CO_2_ prior to and during the addition of 100 mL 10% rumen fluid inoculum. The bottles were back filled with CO_2_ and sealed with butyl rubber bungs. Bottles were then placed in an incubator at 39 °C in the dark. Measurements of total gas production, CO_2_ and methane were taken at time intervals of 0, 4, 8, 12, 18, 24, 30 and 48 h. For each sampling point, the head space of the bottles was collected and measured into a 50 mL syringe, informed by a pressure transducer (Bailey and Mackey Ltd, Birmingham, UK)^45^. The gas composition was analysed by an IRGA (Infra-Red Gas Analyser, ADC, Bioscientific Ltd, UK)^45^. At the end of the time course (48 h) the contents of the serum bottles were strained, and the remaining grass solid constituents were flash frozen in liquid nitrogen for FTIR analysis and following further freeze drying for dry matter degradation. The pH of the rumen inoculum was measured for each bottle and two aliquots of 1 mL rumen inoculum was sampled for measurements of volatile fatty acid (VFA) and ammonia levels. Samples of the rumen inoculum were also used for end point FTIR analysis in order to explore differences in metabolite production due to the fermentation of the forages incubated at a system level.

#### Volatile organic compound (VOC) analysis

Non-targeted analysis of volatile organic compounds (VOCs) was used to assess entire gaseous fermentation profiles in addition to targeted measurements of the major volatile fatty acids produced by rumen fermentation (acetate, butyrate, propionate). VOCs were collected from the head space of the serum bottles 24 h after the start of in vitro fermentation using inert coated SafeLok tubes for Odour/Sulphur analysis (Markes International, Llantrisant, UK) fitted to the IRGA. Tubes were dry purged prior to sample desorption for 1 min with 20 mL/min nitrogen at room temperature. Tube desorption (primary desorption) was conducted by thermal desorption using a TD 100 (Markes International, Llantrisant, UK) for 10 min with 40 mL/min at 280 °C to trap at 25 °C. Trap desorption was then conducted for 6 min with 6.5 mL/min helium at 300 °C (max heating rate) 1.5 mL/min onto the column (split ratio 1:4.3). Samples were then re-collected onto sample tubes. Gas Chromatography (GC 7890A, Agilent Technologies) was conducted initially at 40 °C for 4 min followed by 4 °C/min to 250 °C then 20 °C/min to 300 °C and final hold for 2 min (62 min total). Separation was done over 60 m × 0.32 mm × 0.5 mm MEGA-5 MS with 1.5 mL/min helium under a constant flow condition. Mass spectrometry was carried out using a BenchTOFdx (Markes International, Llantrisant, UK) in EI + mode. Spectra were recorded from m/z 35 to 450 (at 1970 scans/scanset) with a transfer line temperature 250 °C and an ion source temperature of 250 °C. A retention time standard (C8–C20, Sigma Aldrich) was used, injecting 1 μL onto a collection tube (Tenax TA, Sulficarb) and analysed as described for the samples.

#### Chemical analysis

Samples for VFA analysis were acidified by the addition of 4% orthophosphoric acid (final concentration)^46^ and analysed by gas chromatography with 4 mM ethylbutyric acid as an internal standard^47^. Ammonia was analysed from end point samples by acidifying with the addition of concentrated hydrochloric acid (5% v/v final concentration)^48^. Aliquots of incubation buffer and rumen inoculum were analysed by FTIR (Equinox 55 HTS-XT FTIR Spectrophotometer, Bruker UK Ltd, Coventry, UK), where 10 μL of sample was spotted onto a 96 well silicone plate dried to 40 °C before scanning. Remaining plant material was treated as with initial plant material prior to in vitro batch fermentation. All samples were analysed individually with 10% of the samples analysed again for quality control purposes. The FTIR spectra obtained were converted to XY data. The generation of XY data matrices from FTIR analysis were exported in ASCII format. The spectra were mined^49^ and data were examined using principal components analysis (PCA) using Pychem software^50^.

#### Grass physiological characteristics and gas production analyses

Data analysis was conducted in R version 3.6.0 using RStudio^51,52^. Packages *agricolae*^53^, *car*^54^, *ggplot2*^55^, *lsmeans*^56^ and *rcompanion*^57^ were used. All data were analysed using 2-way ANOVA, further using Tukey mean separation, with the exception of the gas production data. These were analysed using general linear models; in each case, the response was log-transformed to meet parametric assumptions, and the predictors were grass variety, climate scenario, the variety-climate interaction, starting mass of dry matter (DM) and biological replicate number. Pairwise comparisons were performed using least-squares means with Tukey adjustment. Biological replicate number was included in the model to account for non-independence between samples taken on the same date. It was also necessary to include the starting mass of dry matter, as this inevitably varied slightly between samples. The relationship between dry matter and gas production changes over time, such that simply expressing the data as ml gas/g DM does not accurately account for the correlation as fermentation progresses. Instead, using dry matter as an independent variable allows the model to estimate and control for the effect of dry matter at the time of sampling (48 h). The model predictions were obtained for all samples based on 1 g DM.

#### Analysis of VOC data

Initial deconvolution and peak identification were carried out in AMDIS v2.72, using the NIST database v2.2 (2014). A custom compound library was built using a training dataset of one sample from every treatment-variety combination, distributed across biological replicates. Components were included in the library if they hit against the database (score > 80) and a retention index (RI) ± 30 the database value. Where the top compound produced a convincing spectrum match but fell outside the RI range, the component was added to the library and named by chemical group, e.g. Acid1. All samples were then run against this library with settings as recommended by Ref.^58^. The AMDIS results were validated and backfilled in Gavin 3.97^59^ using the parameters given in Table S1; raw outputs from Gavin are given in Table S2. All data analysis was carried out in R version 3.5.2 ‘Eggshell Igloo’ using RStudio^51,52^ and packages *metacoder*^60,61^, *mvabund*^62^, *randomForest*^63^ and *vegan*^64^. R code to reproduce the analyses is available as a Rmarkdown file at https://github.com/ecologysarah/lolium-rumen-voc^65^. Known contaminant compounds were removed from the peak table, along with compounds corresponding to those present in machine blanks and any compounds that did not occur in all three replicates of at least one treatment-variety combination. Peaks were normalised to percentage area per sample, to account for differences in intensity. Normalised peak data was log10 transformed to meet parametric assumptions, and modelled using a multivariate linear model (*manylm*^62^ with grass variety, climate scenario, biological replicate number and the variety-climate scenario interaction as predictors. Pairwise comparisons were conducted by splitting the data into subsets, running *manylm* on each one, and adjusting the *P* values with the Benjamini–Hochberg correction^66^. Data were visualised by organising the compounds into hierarchical groups and plotting heat trees^60^. Random Forest™ analysis was undertaken to assess classification of data by variety, treatment or experiment, and to identify which compounds were most informative for classification^67^. The proximity scores created in the process were used as a distance matrix for multi-dimensional scaling (MDS) to plot the outputs. For each forest, the 25 compounds that contributed the largest mean decrease in accuracy when excluded) with a z-ratio ≥ 7 were extracted from the data and used to create a new random forest.

## Results

### Effect of growth conditions on physiological characteristics of grass varieties at harvest

Overall, the grass varieties were clearly affected by the climate scenarios imposed during growth, indicating that the extent of the stress imposed was sufficient to elicit a physiological response (Fig. S1). Mean biomass values for grasses in each climate scenario (control 2020, control 2050, 2050 + flood, 2050 + drought and 2050 + heat shock) were 14.4 g, 10.2 g, 8.6 g, 6.0 g and 5.8 g DM respectively (SD = 3.61). There was no significant effect of grass variety on Fv/Fm ratio (*P* = 0.328), chlorophyll (*P* = 0.118) or crude protein (*P* = 0.702), with mean values of 0.79 (Fv/Fm), 8.86 ug/mL and 23.8% respectively (Supplementary Table S1). However, there was a significant effect of grass variety on water-soluble carbohydrate (WSC) (*P* = 0.030), with Aber Niche having a higher (*P* = 0.031) WSC compared to Aber Root, although there were no differences amongst any of the other grass varieties. The fibre values for NDF, ADF and ADL varied (P < 0.001) between grass varieties, however the mean values were 38.7%, 15.3% and 2.2% respectively (Table S1). Taking all the varieties together, there was an effect (P < 0.05) of climate scenario on crude protein, WSC, chlorophyll, Fv/Fm ratio, NDF, ADF and ADL (Table 3).

**Table 3:** Mean values for dry matter% (DM) crude protein (as a % of DM), water soluble carbohydrate (WSC as a % of DM), chlorophyll content, Fv/Fm ratio NDF, ADF and ADL in each climate scenario.

Analysis	2020	2050	2050 + flood	2050 + drought	2050 + heat shock	S.D.
Fv/Fm	0.81^b^	0.81^b^	0.80^ab^	0.78^ab^	0.76^a^	0.05
Chlorophyll	11.12^c^	9.08^ab^	6.19^a^	10.05^bc^	7.84^ab^	2.90
WSC %DM	4.24^b^	3.04^ab^	2.57^a^	3.52^b^	3.64^b^	1.17
Crude protein% DM	23.2^ab^	22.5^a^	21.6^a^	26.7^b^	25.0^ab^	3.72
Dry matter%	15.6^bc^	13.1^ab^	10.2^a^	18.3^c^	15.7^bc^	3.42
NDF %DM	36.0^a^	40.0^b^	44.1^c^	37.4^a^	37.5^a^	4.5
ADF %DM	12.8^a^	16.4^d^	18.0^e^	15.0^c^	14.4^b^	3.6
ADL %DM	2.3^c^	2.4^d^	3.3^e^	1.2^a^	2.0^b^	1.2

Lower case letters indicate significant difference based on ls means with Tukey adjustment (*P* < 0.05).

**Figure 1:**
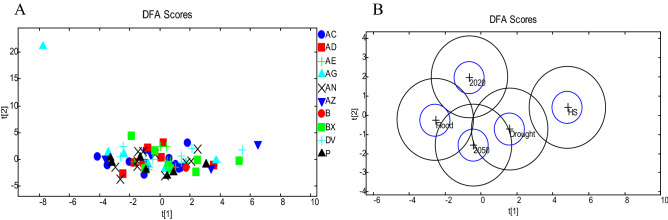
DFA analysis of grass variety based on FTIR analysis, showing no distinct clustering between varieties (*AC* Aber Clyde, *AD* Aber Dart, *AE* Aber Echo, *AG* Aber Glyn, *AN* Aber Niche, *AZ* Aber Zeus, *B* Barolex, *BX* Aber Root, *DV* Da vinci, *P* Premium (**A**); climate scenarios (**B**), showing distinct clustering between environmental conditions (DFA circles around the mean group centres with 95% confidence circles for DF1 vs DF2; *HS* heat shock).

Discriminant functional analysis (DFA) of Fourier Transform infra-red spectroscopy (FTIR) spectra to determine differences in the plant chemistry at harvest showed no clustering by grass variety. However, clustering was observed for climate scenario, with heat shock FTIR spectra differing from all remaining scenarios except drought (Fig. 1).

### Cumulative gas production

**Figure 2:**
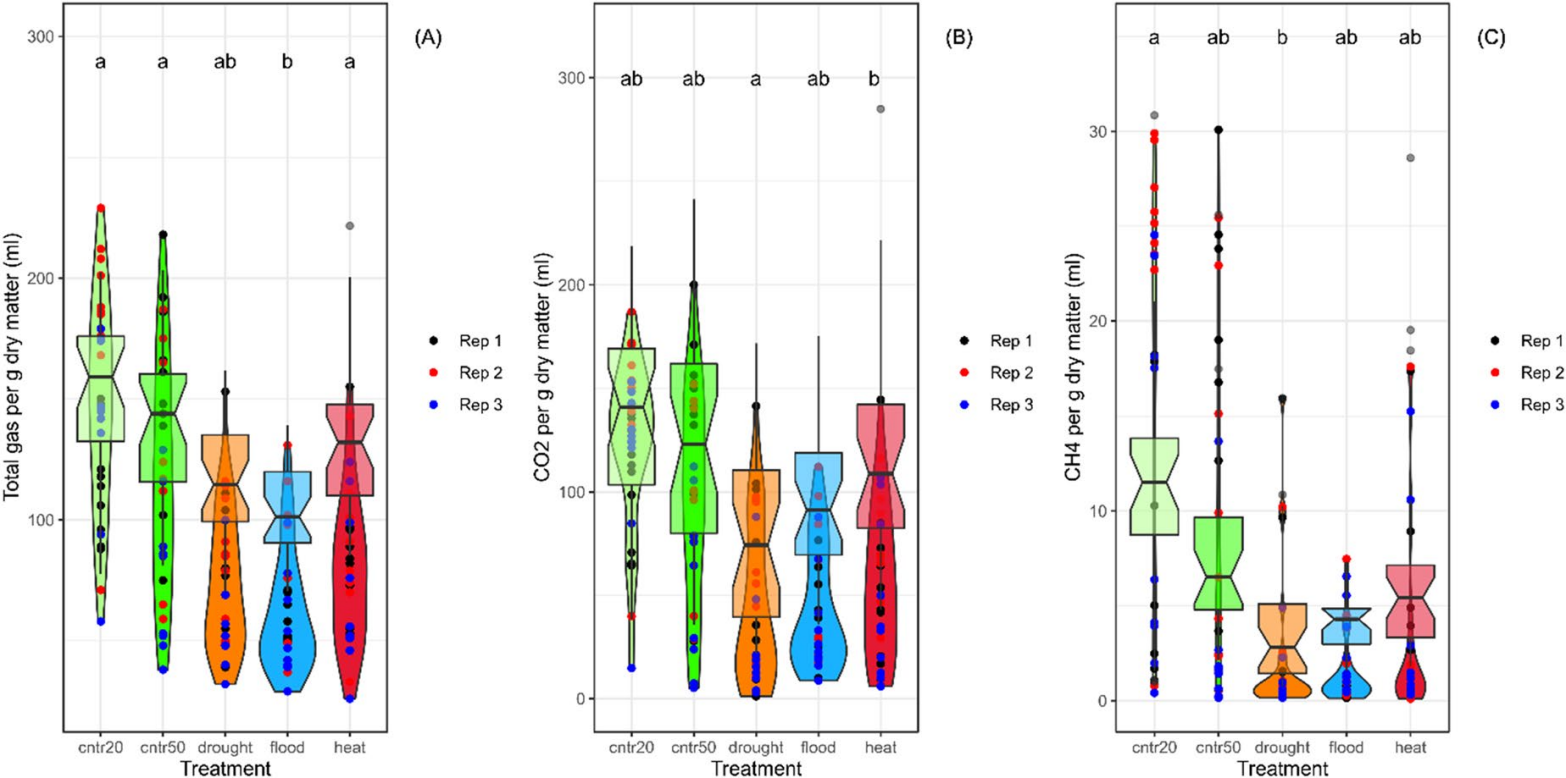
Effect of climate scenario on total gas production (**A**), total CO_2_ production (**B**) and total methane production (**C**) at 48 h fermentation as affected by climate change scenario (all corrected for starting weight of dry matter); *HS* heat shock). Raw data points are overlaid on boxplots, coloured by replicate experiment. Notches represent 95% confidence intervals. Lower case letters indicate significant difference based on ls means with Tukey adjustment (*P* < 0.05).

Total cumulative gas production measurements were taken 48 h from the start of the in vitro rumen fermentation experiments (Fig. 2A): a greater amount of gas produced during the 48 h indicates more extensive fermentation. There was a significant effect of grass variety (*P* = 0.041) on gas production, driven by lower gas production in the Aber Zeus samples (Fig S2). There was a significant effect of climate on gas production (*P* = 0.002), and pairwise comparisons showed that the flooding treatment produced significantly less gas than the 2020 controls (*P* = 0.014), 2050 controls (*P* = 0.038) and the heat shock treatment (*P* = 0.016) (Fig. 2A). There was no significant interaction between variety and climate scenario.

There was no effect of grass variety on CO_2_ production (*P* = 0.114), nor any significant interaction between variety and climate scenario. However different climate scenarios did result in differences (*P* = 0.016) in CO_2_ production which was lowest in the drought treatment compared to heat shock (Fig. 2B). Methane (CH_4_) production during in vitro fermentation showed no effect due to grass variety (*P* = 0.111), nor any significant interaction between variety and climate scenario. However, growth under different climate scenarios did have an effect on methane production (*P* = 0.016; Fig. 2C), which was again lowest in the drought scenario compared to control 2020.

### In vitro rumen fermentation parameters

**Figure 3:**
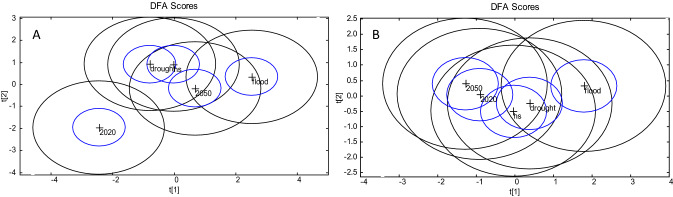
DFA analysis of liquid (**A**) and pellet (**B**) FTIR as affected for each climate scenario at the end of 48-h fermentation (DFA circles around the mean group centres with 95% confidence circles for DF1 vs DF2; *HS* heat shock).

FTIR analysis at the end fermentation after 48 h showed a separation of clusters for the liquid fraction with the flood treatment resulting in differentiation from 2020 climate condition (Fig. 3A). However, there was no differentiation of clusters within the pellet (Fig. 3B).

There was no effect of grass variety on total VFA production (*P* = 0.401), ammonia production (*P* = 0.580) or pH (*P* = 0.700) with mean values of 251 mM/g DM, 43 mg/g DM and 6.8 respectively. However, climate change scenario affected fermentation profiles of all the parameters analysed (*P* < 0.001; Table 4).

**Table 4:** Fermentation profile values for VFA, ammonia and pH end point analysis.

	2020	2050	2050 + flood	2050 + drought	2050 + heat shock	S.D.
DMD g/100 g	77.5^a^	74.2^ab^	55.4^c^	54.6^c^	63.3^bc^	18.0
VFA mM/g DM	315^a^	284^ab^	229^bc^	202^c^	227^bc^	51.6
Ammonia mg/g DM	28^b^	32^b^	52^a^	51^a^	53^a^	26.6
pH	6.7^a^	6.8^a^	6.9^b^	6.9^b^	6.9^b^	0.11
Acetate mM/g DM	174^a^	166^a^	148^a^	109^b^	145^a^	26.4
Propionate mM/g DM	66^a^	58^ab^	40^c^	41^c^	43^bc^	15.6
Isobutyrate mM/g DM	5^a^	4^ab^	3^b^	3^b^	3^b^	1.0
Butyrate mM/g DM	47^a^	38^ab^	25^b^	35^a^	23^b^	9.7
Acetate: propionate	2.8^a^	3.1^ab^	3.9^c^	2.9^a^	3.6^bc^	0.86

Lower case letters indicate significant difference based on ls means with Tukey adjustment (*P* < 0.05).

There was no difference between plant variety Dry Matter Degradation (DMD), mean value of 65.0 g/100 g, however climate scenario significantly affected DMD with future scenarios, flood, drought and heat shock, being significantly less than control 2020 (Table 4). Climate scenario affected VFA production with there being no difference between control 2020 and control 2050 but all other treatments resulted in a lower (P < 0.05) value compared to control 2020. Ammonia concentration was lowest (P < 0.05) in control 2020 and control 2050 compared to all other climate scenarios. The pH in the 2020 and 2050 controls was lower than in all other climate scenarios, however this small change is considered not biologically significant. Acetate production was lowest (P < 0.05) in 2050 + drought compared to all other climate scenarios, whereas propionate concentration was highest (P < 0.05) in control 2020 with decreasing values for control 2050, 2050 + heat shock, 2050 + drought and 2050 + flood respectively. The iso butyrate concentrations were different (P < 0.05) between climate scenarios but a difference of 2 mM was again considered not biologically significant. Butyrate concentration was highest (P < 0.05) in control 2020 and 2050 + drought and lowest in 2050 + flood and 2050 + heat shock with an intermediate value for control 2050. The acetate to propionate ratio (C2:C3) was lowest (P < 0.05) in control 2020 and 2050 + drought and highest in 2050 + flood with intermediate values for control 2050 and 2050 + heat shock.

### Volatile organic compounds (VOCs)

Across the entire dataset, 175 distinct VOCs were detected. Climate scenario was a significant predictor of VOC profiles overall (LR_4,134_ = 5372, *P* = 0.002), and all pairwise comparisons were also significantly different from each other (*P* < 0.05) (Table 6).

**Figure 4:**
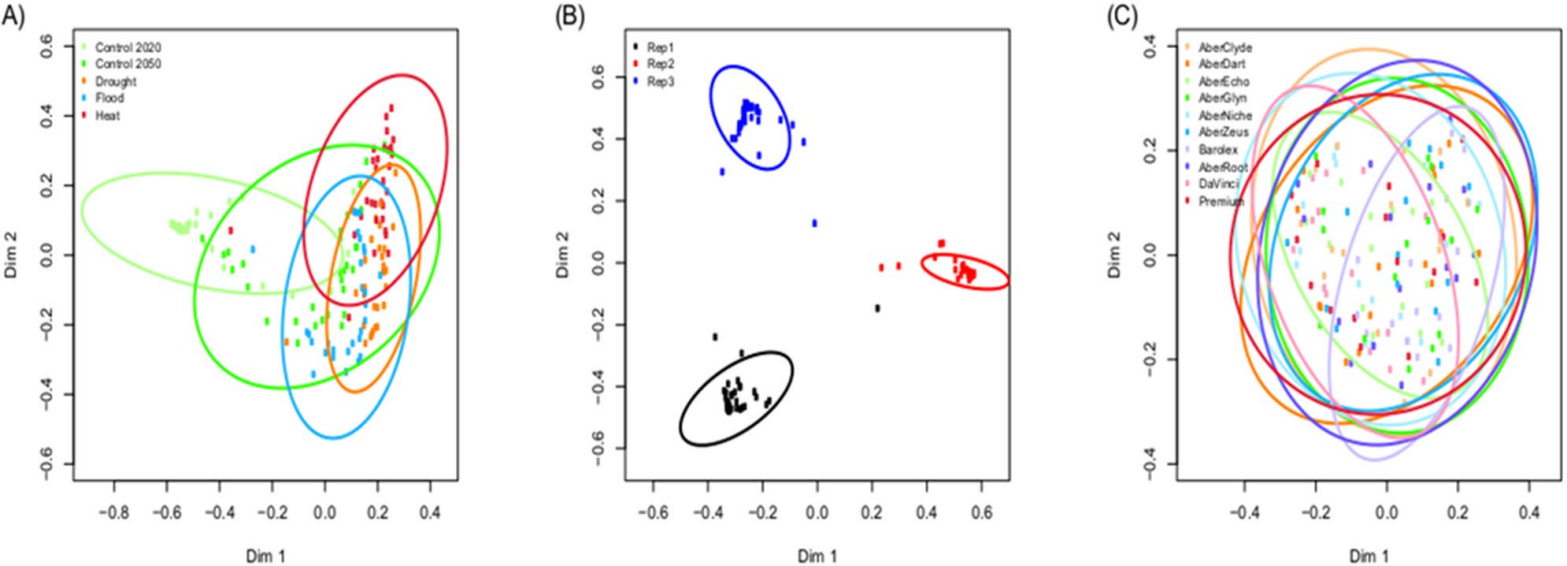
MDS ordination of VOC profiles of grass varieties exposed to rumen fluid, based on proximity values from random forest classification by (**A**) climate scenario; (**B**) biological replicate; and (**C**) variety of grass.

Random forest analysis correctly classified at least 66% of samples in each climate scenario, despite only partial separation on the MDS plot (Table 5, Fig. 4A). Climate scenarios could be successfully differentiated despite marked and significant differences between biological replicates (LR_2,134_ = 9833, *P* = 0.002; Table S4; Fig. 4B). No grass varieties could be separated from each other (Fig. 4C).

**Table 5:** Results from random forest classification of VOC samples by growth condition at 24 h post incubation.

		Predicted class					Error rate (%)
		Control 2020	Control 2050	2050 + drought	2050 + flood	2050 + heat shock	
True class	Control 2020	**26**	4	0	0	0	13
	Control 2050	1	**23**	1	2	3	23
	Drought	0	3	**21**	4	2	33
	Flood	1	2	2	**24**	1	20
	Heat	1	0	5	0	**24**	20

Rows represent the number of samples from each true class that were allocated to each of the predicted classes (columns). The error rate is the percentage of samples that were misclassified from each group. The predicted class for each given sample was determined by a simple majority of votes from the trees in which that sample was out-of-bag (OOB).

Correct classifications are shown in bold.

Random forests using subsets of only the 25 most discriminatory compounds performed comparably to the originals in their respective classifications (Table 6, Table S4), indicating that these subsets captured much of the relevant information from the full dataset. Within each growth condition, the most important compounds in classification were not necessarily the most abundant and vice versa (Table 6). Only seven compounds (2,4-dimethylfuran, butan-2-one, 3-methylbutan-1-ol, 3-methylpentan-2-one, methylsulfonylmethane, heptane and 1,1'-oxydibenzene) were shared between the discriminatory subsets for climate scenario and biological replicate, further indicating that the effects of climate are robust to background variability (Table S3).

**Table 6:** Top 25 compounds most important for random forest classification of climate scenario, ranked in order of their importance in discriminating each climate scenario at 24 h post incubation (see Table S2 for raw importance scores).

	Control 2020		Control 2050		2050 + drought		2050 + flood		2050 + Heat shock	
	Abundance	Rank	Abundance	Rank	Abundance	Rank	Abundance	Rank	Abundance	Rank
(2Z,4E)-hexa-2,4-diene	0.0207	13	0.00981	16	0.00573	6	0.00661	7	0.00612	9
Octa-1,3-diene	0.0161	5	0.0532	2	0.0499	5	0.0165	16	0.022	14
3-Ethylocta-1,5-diene	0.0172	14	0.0109	15	0.0115	7	0.0219	9	0.008	8
Pentan-3-ol	0.156	3	0.0652	6	0.0251	16	0.0298	13	0.0168	15
Methanethiol	0.386	4	0.296	3	0.152	17	0.2	3	0.0959	12
Penta-1,3-diene	0.116	1	0.0463	8	0.0149	14	0.0279	18	0.0114	5
2,4-Dimethylfuran	0.237	6	0.15	7	0.11	9	0.12	15	0.0878	4
Acid1	0.0217	2	0.0272	23	0.0428	19	0.0471	5	0.0441	3
Pent-1-en-3-ol	0.0841	9	0.0392	9	0.0218	4	0.0249	19	0.0152	1
Dodecane-1-thiol	0.00961	10	0.0113	10	0.0152	3	0.016	20	0.0149	2
Methylsulfonylmethane	1.07	8	1.82	11	1.2	1	1.46	8	1.38	17
2-Ethylthiophene	0.0499	18	0.0669	1	0.0421	18	0.0392	1	0.0353	16
1,1′-Oxydibenzene	0.0412	16	0.0528	18	0.065	8	0.0723	12	0.0626	7
2-Methylbut-1-ene	0.563	11	0.257	24	0.144	2	0.247	10	0.104	6
Ethanol	2.02	15	2.27	20	1.49	13	1.49	6	1.15	10
3-Methylpentan-2-one	5.68	17	4.97	5	4.93	20	4.32	11	5.46	13
Butan-2-one	5.28	20	4.72	13	4.69	10	4.13	4	5.2	19
Decanal	0.0546	21	0.0511	22	0.0927	22	0.0844	2	0.0863	20
3-Methylthiophene	0.639	23	0.629	12	0.195	15	0.253	14	0.284	11
(3E)-1,3-hexadiene	0.0192	24	0.0227	17	0.0122	21	0.0115	22	0.0112	21
Dimethyltrisulfane	0.777	7	0.903	4	0.658	12	0.899	17	0.371	24
3-Methylbutan-1-ol	0.0245	22	0.0281	25	0.0204	23	0.0252	21	0.0172	23
(Z)-oct-2-ene	1.37	12	1.61	14	1.71	11	1.4	25	1.57	25
Heptane	0.822	19	0.944	21	0.648	25	0.35	23	1.23	18
(E)-oct-4-ene	1.38	25	1.61	19	1.7	24	1.4	24	1.57	22

Based on 100,000 trees. Abundance is the mean relative abundance of the compound in each growth condition (based on total area normalisation). Compounds are shown in order of decreasing importance to the overall classification (the relative reduction in random forest performance when the values for a given compound are randomly permuted). Rank represents order of importance for each growth category.

**Figure 5:**
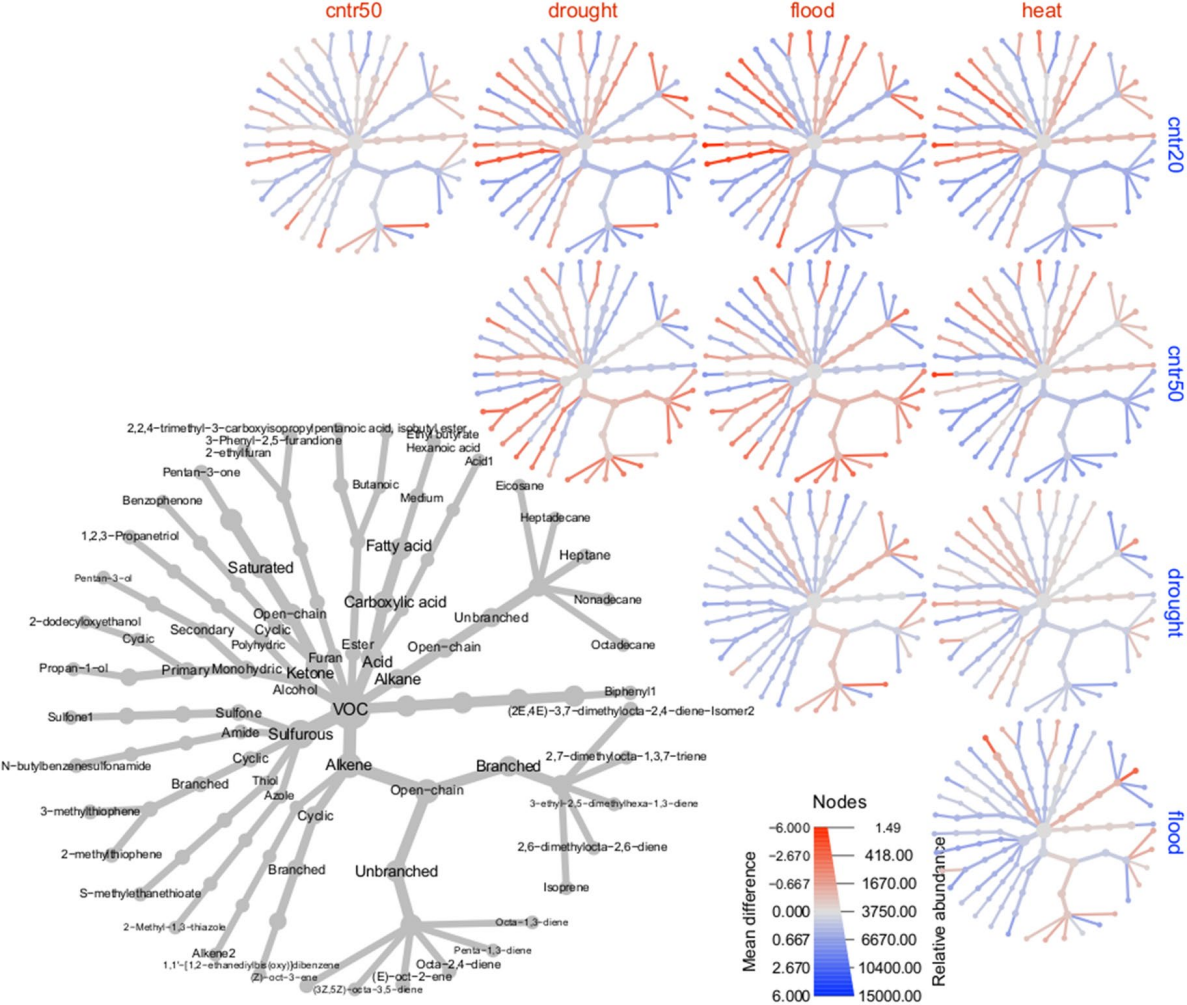
Comparisons between VOC profiles from grass varieties exposed to rumen fluid, broken down by climate scenario. Compounds are classified into hierarchical classes, and every node represents on the heat trees one compound or parent class. Node size is proportional to relative abundance for that class/compound. Each of the small heat trees represents a comparison between two climate scenarios. Node colour is on a scale representing the mean abundance change between conditions as a proportion of the total abundance of that compound: orange indicates that the compound had higher relative abundance in the ‘column’ treatment, grey indicates no difference, and blue indicates higher relative abundance in the ‘row’ treatment. Relative abundance is on an arbitrary scale where each sample sums to 100. For ease of viewing, compounds are only included if their relative abundance at least doubled in at least one comparison. For this reason, in some cases a node will show a different direction of change to its parent nodes (i.e. other compounds, not included in the plot, contribute to the parent node’s direction of change).

By relative area (normalised per sample), the most abundant group was sulphurous compounds (33% of total area), followed by alkenes (20%), acids (11%) and ketones (11%) (Fig. 5, main panel). By number of compounds, the largest group was alkenes (47 compounds), followed by sulphurous compounds (24 compounds) and alcohols (20 compounds). The relative abundance of alkenes decreased in the future scenarios compared to the 2020 control, while acids increased (Fig. 5).

## Discussion

### Grass physiological changes in current and future environmental conditions

Climate change involves multiple parameters which individually and in combination can alter plant physiology. It is currently unclear if these changes would be beneficial or detrimental to forage quality and hence the impact on ruminant production. During abiotic stresses plants alter their biomass allocation. In this work the lowest total biomass was from the droughted grasses and those subjected to heat stress, which correlates with previous work where biomass allocation decreased with the abiotic stresses of drought and temperature^68^. Water soluble carbohydrate levels (WSC) in the control 2020, 2050 as well as the drought and heat shock climate scenarios were higher than the flood scenario, which correlates with previous work where drought and heat stress conditions have been shown to generally elevate WSC as a part of a protective mechanism^28^. However, the combination of drought with elevated levels of CO_2_ may also have an effect on the drought response resulting in slightly higher WSC and dry matter levels^25^. Flooding resulted in a negative effect on crude protein levels and water-soluble carbohydrate levels as might be expected, due to reduced photosynthetic capacity and increased the proportion of fibre. However, protein concentrations were found to be in higher in the drought and heat shocked grasses than in controls, in contrast to previous studies^69,70^. Drought stress reduces photosynthetic activity and Rubisco activity^71^ and hence lowers protein levels. However, these effects have been demonstrated not to occur until severe or long-term drought stress has occurred^72,73^ and indeed in the experiments reported here the chlorophyll level and Fv/Fm were not significantly affected by the duration of 2050 drought treatment. The ratio of Fv/Fm showed a significantly lower value for grasses subjected to the heat shock climate scenario. This was found to be below the optimal value for most plants, indicating plant stress by the photoinhibition of photosystem II^74^. Chlorophyll measurements can also indicate how photosynthesis is affected during stress^75^. Chlorophyll levels were highest in the control 2020 scenario and the lowest in grasses subjected to heat shock and flood climate scenarios, indicating that photosynthesis was affected by both these treatments, which is consistent with previous studies^26,32,33,75^.

Again, the elevated CO_2_ imposed together with the drought, as well as the short drought period may have mitigated the negative effects on protein content. The clustering of the FTIR data based on treatment further indicates that the treatments were sufficient to elicit a response, and the separation of the heat-shocked material from the controls fits with the reduced chlorophyll and Fv/Fm elicited for this treatment indicating a high level of imposed stress.

### Effects of climate condition of grass growth on rumen fermentation

Growth of these grasses under different climate conditions was shown to have an impact on fermentation. It is suggested that the plant response to climate has a consequence for the activity of the rumen microbiota; altering the nutrient provision to the colonising microbiota affects colonisation community profiles and in consequence, forage degradation parameters^12,76^. This is likely to be a result of differences in forage chemistry due to acclimation responses during growth but could also be a result of changes caused by active plant stress responses to the rumen environment, as has been previously demonstrated to occur in grass and clover^16–18,76^. The effects of pre-harvest climate scenarios on rumen fermentation are in agreement with previous work indicating that plants have a ‘stress memory’ or ‘defence priming’ and that stress responses are influenced by previous exposure^34,77,78^. The in vitro gas production technique is a method for analysing fermentation of feeds by a rumen microbial inoculum. The analysis of loss of dry matter (DM) and amount of gas (CO_2_ and CH_4_) produced as a result of fermentation of the grass feed indicated that the growth conditions could affect how the grasses would be digested by the animal. Flood had the greatest effect in reducing total gas production, compared to both the control climate scenario for 2020 and 2050. This is probably linked to the decreased WSC and increased NDF content of flooded grasses compared with those grown under 2020 or 2050 conditions. This suggests that breeding forage grasses for resilience to a future climate in which there are periods of flooding during the growth period may be important for maximising its nutritional value.

The in vitro DMD values observed in this study for control 2020 are typical of those expected in high quality pastures for ruminants^79^. The reduced in vitro DMD caused by future climate scenarios, especially periods of flooding or drought, would lead to reduced animal performance and would require higher intakes to maintain the level of production^80,81^. Interestingly, methane levels were lower from fermentative grasses that had been previously subjected to flood, drought or heat shock compared to the 2020 and 2050 controls, potentially due to physiological damage caused by flood, drought and heat shock. Different silages have been shown to affect rumen fermentation and the microbial community due to differences their chemical composition^82^. For example, methane production was higher in grass silage compared to corn silage and feeding ruminants with a more digestible forage reduced methane production^79,83,84^. Thus, the lower levels of methane produced from grass subjected to 2050 + drought, in this study combined with the lower DMD indicate that the stress treatment affected in vitro rumen fermentation compared to the current climate model. Based on the model-predicted data, the acetate: propionate ratio was 3.26 and the propionate: butyrate ratio was 1.54. This closely matched the expected values of 3.25 and 1.33, respectively^85^. Hydrogen production and therefore methanogenesis was suggested to be altered by the partitioning of degraded DM between microbial synthesis and rumen fermentation^86^. The manipulation of the level of carbohydrate from feed that goes directly into microbial growth rather than fermentation can alter methanogenesis by the hexose partitioning of the feed^87^. The volume of methane production in vivo has been shown to be lowest when animals were fed high sugar diets^88^. Further work confirmed in vivo that partitioning of hexose into fermentation end products (including methane) and microbial biomass is influenced by dietary carbohydrate and by increasing the proportion of WSC in the diet can significantly reduce the amount of methane produced^89^. Although decreased methane production has been linked to increased WSC^89^, this does not explain the results obtained in the present study since WSC remained relatively unaffected by the pre-harvest stress treatments.

FTIR profiles after 48 h of digestion discriminated between plants grown in the flood climate scenario compared to the control 2020 but did not discriminate amongst the different grass varieties. This is in contrast with previous work^2^ where variety did influence the FTIR after 24 h of fermentation. However, it is possible that differences observed after 24 h were not visible after 48 h because of a more extensive degradation by that time. Typically, in fresh forages an increase in acetate is highly correlated to increased methane production, however here while acetate increased, methane was reduced. However, due to the lower DMD values of the stressed forages this may not be reflected in the animal hence further investigation is required.

### Changes in VOC profiles during rumen fermentation due to differing climate conditions

This is one of the first experiments analyse VOCs in relation to plant material under simulated rumen conditions, and it showed that it is possible to distinguish an effect of plant growth conditions from rumen VOCs. The unexpected differences between biological replicates most likely represent differences in the rumen fluid on each occasion and suggests that the exact composition of rumen fluid is a key influence on the volatile bouquet. Despite efforts to keep the replicates as consistent as possible, rumen fluid is affected by diet, health, time since last feed and stochastic changes in microbiota^10,90^ although here animals were on the same diet, fed at the same time, and rumen fluid was also collected at the same time. Thus, changes in VOCs must be affected by very small changes in diet composition or animal physiology. That the effects of stress growth condition could nonetheless be picked out from this high background variability indicates that there are consistent patterns associated with different plant growth conditions. Although none of the ten varieties of grass could be reliably differentiated from the rest, this does not preclude the possibility that a larger sample size would be able to detect subtle differences amongst varieties.

The most abundant VOCs in fresh *L. perenne* have been reported as benzeneacetaldehyde, 2,5-dimethyl-pyrazine, hexanal and benzaldehyde^91^. Of these four, all but 2,5-dimethyl-pyrazine were detectable in the present samples, indicating that grass volatiles could be clearly distinguished in the rumen. In total, eleven of the 58 VOCs listed in Ref.^91^ were also identified here, and for a further seven, closely related compounds were present (i.e. different isomers or branching patterns). In addition, the samples contained 18 of the 50 compounds previously listed in rumen headspace^90^ with another 10 close matches. This included nine of the 16 compounds which were highlighted as major rumen fermentation products or characteristic components of rumen odour: acetic acid, propanoic acid, pentanoic acid, hexanoic acid, dimethyl sulphide, dimethyl disulphide, dimethyl trisulphide, phenol and 3-methyl-thiophene^90^. There was a prevalence of dienes in the present study: 16 in total, of which five were among the 25 compounds most discriminatory between treatments. This abundance of unsaturated compounds is indicative of the reducing environment within the rumen.

Eight of the 25 VOCs identified as major discriminators across the stress growth conditions (Table 6) have been previously associated with plant stress responses. The 3-pentanol activates pathogen defence responses via salicylic acid, jasmonic acid and ethylene signalling pathways^92,93^. The 1,3-octadiene is negatively associated with drought in flowers^94^, while 3-ethyl-1,5-octadiene is an autolysis product and is possibly linked to herbivore defence^95,96^. It is known that 1-penten-3-ol is produced following stresses such as wounding, drying or freezing^97,98^ and has been associated with mown grasslands^99^. It possesses anti-fungal properties^100^, but its formation is oxygen-dependent^101^, suggesting that it was produced prior to rumination. Ethanol, another of the key discriminatory VOCs, is produced by plant cells when anoxia forces them to switch to fermentative metabolism^20^. Ethanol is also produced by microbial fermentation but is a minor component in the healthy rumen^102^; it constituted on average 1.7% total area in the present samples. The compound 3-methyl-2-pentanone is associated with herbivore defence^103^ while 2-ethylthiophene was negatively associated with increased CO_2_ levels in broccoli^104^. Also, 2-butenal is a discriminatory compound worth mentioning, even though it was not in the top 25. This is because 2-butenal strongly induces abiotic stress-related transcription factors^105^ but also causes irreversible damage to chloroplasts^106^.

Both 2-methyl-1-butene and 3-methyl-1-butene are produced from the reduction of isoprene under anaerobic conditions^107^. This is of particular interest as isoprene is used as an electron acceptor during acetogenesis and inhibits the production of methane^107^. Thus, the association of these VOCs with the stress growth conditions and the reduction in methane may be metabolically linked. Isoprene is a major component of plant volatile emissions and offers protection from heat stress and oxidative damage^108,109^. Although only 2-methyl-1-butene featured in the top 25 compounds, isoprene and both its breakdown products were important in differentiating the control 2020, drought and heat shock growth conditions. Isoprene was most abundant in the control 2020 growth condition, which would fit with the general pattern of inhibited isoprene production at increased CO_2_ concentrations^109^.

Several of the discriminatory compounds were also associated with microbial metabolism. Methanethiol is produced by the microbial breakdown of S-containing amino acids^110^ and also plays an important role in anaerobic methane cycling^110,111^. The compound 1-butanol, 3-methyl is a by-product of amino acid fermentation^112^. This compound has also been detected in plant leaves^100^ but given that it is produced by various fungal endophytes it is not necessarily endogenous^113,114^, indeed, it is inhibitory to germination and growth in Arabidopsis^114^.

## Summary and conclusions

It is currently not clear what the impact of climate change on rumen fermentation will be. Consideration of the effect of altered environmental conditions on both plant growth and animal physiology is required. Here we have investigated the former, to explore whether forage growth under an altered climate would produce legacy effects in forage that would impact on rumen fermentation when ingested. Although there was relatively little variation between grass varieties, consistent effects due to growth under severe weather events were detected. Notably acute flood and drought caused decreased digestibility in those forages. As these conditions are predicted to increase in frequency over the next decades, unless addressed this will have a limiting effect on production efficiency. The detection of VOCs associated with plant stress responses during fermentation is further evidence that post-ingestion plant metabolism is a component of the functional rumen system. Together, these data indicate that forage breeding strategies should consider response to future as well as current climates to ensure economic and environmental sustainability of production.

## Supplementary Information

Supplementary Information 1.

Supplementary Information 2.
