# Author Correction: Forage grass growth under future climate change scenarios affects fermentation and ruminant efficiency

**DOI:** 10.1038/s41598-022-21958-y

**Published:** 2022-10-31

**Authors:** Elizabeth H. Hart, Sarah R. Christofides, Teri E. Davies, Pauline Rees Stevens, Christopher J. Creevey, Carsten T. Müller, Hilary J. Rogers, Alison H. Kingston‑Smith

**Affiliations:** 1 Institute of Biological, Environmental and Rural Sciences (IBERS), Aberystwyth University, Aberystwyth, Wales, SY23 3FG UK; 2 School of Biosciences, Cardiff University, The Sir Martin Evans Building, Museum Avenue, Cardiff, CF10 3AX UK; 3 School of Biological Sciences, Queens University Belfast, Belfast, BT9 7BL UK

Correction to: *Scientific Reports* 10.1038/s41598-022-08309-7, published online 15 March 2022

The original version of this Article contained errors in Table 1, where the “Type” values of the “Forage grass” were incorrect for “AberDart”, “AberEcho” and “AberGlyn”, “AberNiche”, “AberRoot”, “Barolex” and “Davinci”. The correct and incorrect values appear below.

Correct:

**Table:** 

Forage grass	Type
AberClyde	Tetraploid perennial ryegrass, high sugar (*Lolium perenne*)
AberDart	Diploid perennial ryegrass, high sugar (*Lolium perenne*)
AberEcho	Tetraploid hybrid ryegrass, high sugar (*Lolium × boucheanum*)
AberGlyn	Tetraploid perennial ryegrass (*Lolium perenne*)
AberNiche	Tetraploid festulolium (Italian ryegrass × meadow fescue) (*Lolium multiflorum x Festuca pratense*)
AberRoot	Perennial tetraploid festulolium, high sugar (perennial ryegrass × Atlas fescue) (*Lolium perenne *×* Festuca mairei*)
AberZeus	Diploid perennial ryegrass, high sugar (*Lolium perenne*)
Barolex	Allohexaploid, tall fescue (*Festuca arundinacea*)
Davinci	Diploid Italian ryegrass (*Lolium multiflorum*)
Premium	Diploid perennial ryegrass (*Lolium perenne*)

Incorrect:

**Table:** 

Forage grass	Type
Aber Clyde	Tetraploid perennial ryegrass, high sugar (*Lolium perenne*)
Aber Dart	Diploid perennial, high sugar (*Lolium perenne*)
Aber Echo	Tetraploid hybrid ryegrass, high sugar (*Lolium perenne*)
Aber Glyn	Tetraploid perennial ryegrass, high sugar (*Lolium perenne*)
Aber Niche	Perennial diploid festulolium (Italian ryegrass × meadow fescue) (*Festuca perennis × Festuca pratense*)
Aber Root	Perennial diploid festulolium
Aber Zeus	Diploid perennial ryegrass, high sugar (*Lolium perenne*)
Barolex	Tall fescue (*Festuca arundinacea*)
Davinci	Diploid Italian ryegrass (*Festuca perennis*)
Premium	Diploid perennial ryegrass (*Lolium perenne*)

The original Article has been corrected.

