# Supplementary material for: Forage grass growth under future climate change scenarios affects fermentation and ruminant efficiency
Source: Sci Rep. 2022 Mar 15;12:4454. doi: 10.1038/s41598-022-08309-7 (PMC8924208; doi:10.1038/s41598-022-08309-7)
Supplement: Supplementary file 1 — Supplementary Information 1. [file 41598_2022_8309_MOESM1_ESM.docx]

Supplementary material

| Grass Variety | 2020 | 2050 | Flood | Drought | Heat shock |
| --- | --- | --- | --- | --- | --- |
| Aber Zeus | 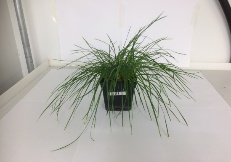 | 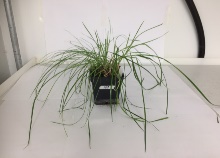 | 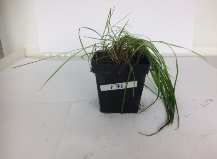 | 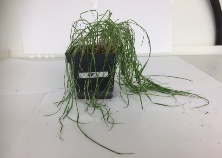 | 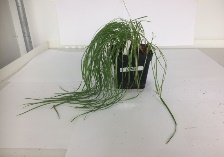 |
| Aber Echo | 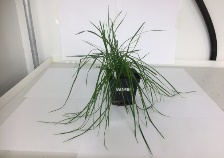 | 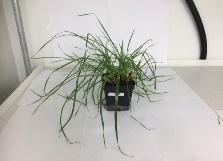 | 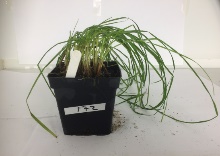 | 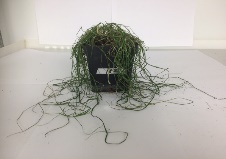 | 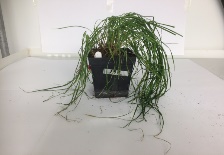 |
| Aber Niche | 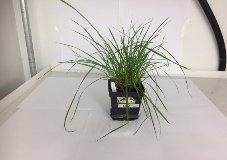 | 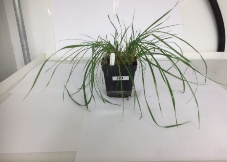 | 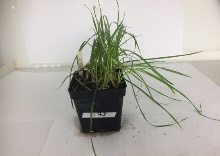 | 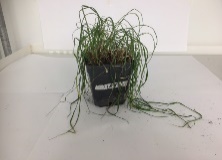 | 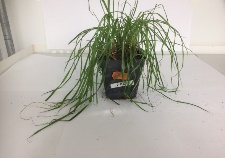 |
| Aber Clyde | 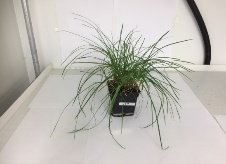 | 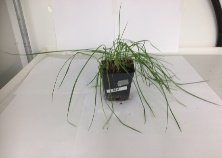 | 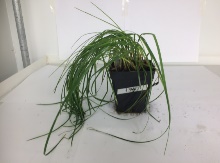 | 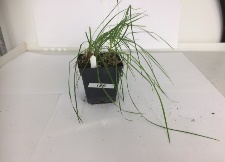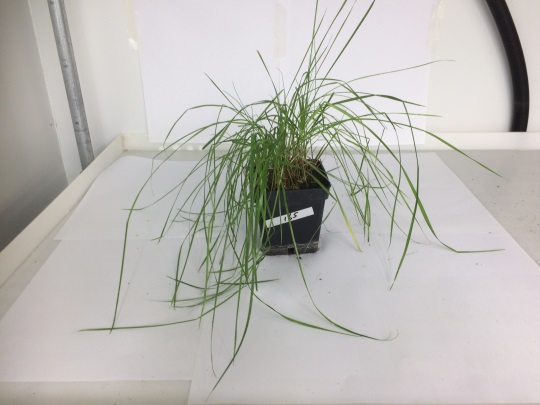 | 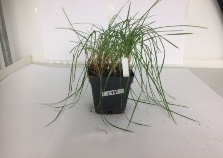 |
| Barolex | 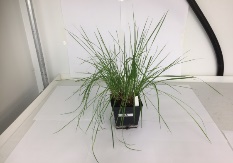 | 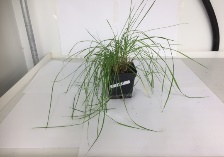 | 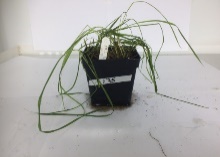 | 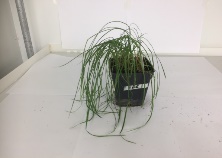 | 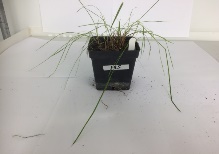 |
| Premium | 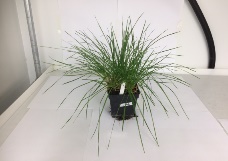 | 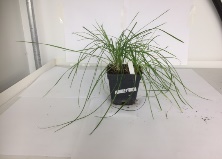 | 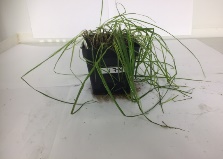 | 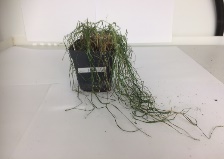 | 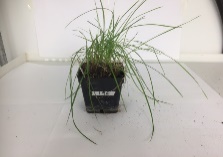 |
| Aber Root | 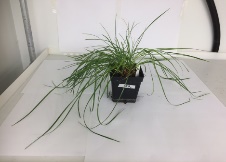 | 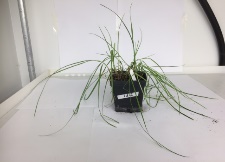 | 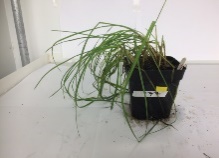 | 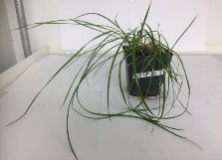 | 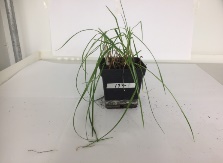 |
| Aber Dart | 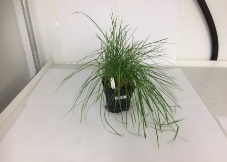 | 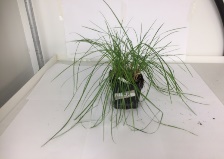 | 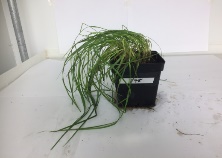 | 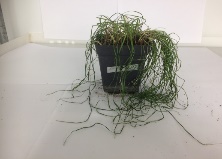 | 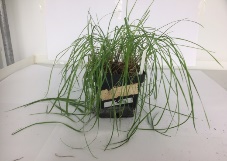 |
| Da Vinci | 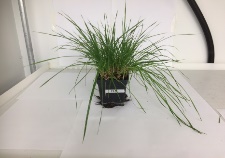 | 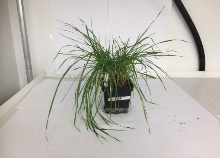 | 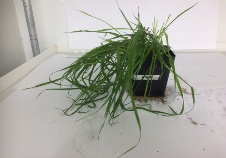 | 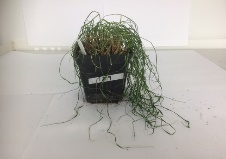 | 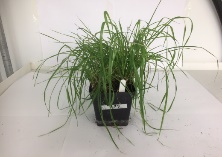 |
| Aber Glyn | 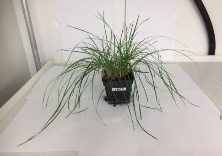 | 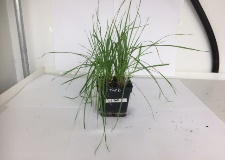 | 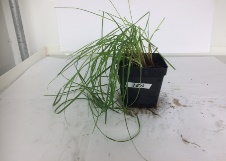 | 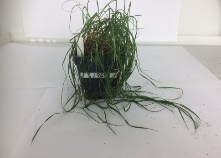 | 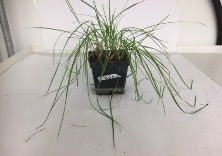 |

Figure S1. Photographs of each grass type phenotype in its corresponding climate scenario prior to harvest.


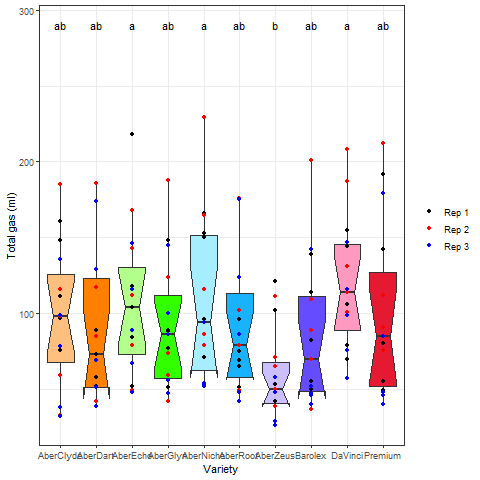


Figure S2. Effect of grass forage variety on total gas production at 48 hours fermentation (all corrected for starting weight of dry matter). Raw data points are overlaid on boxplots, coloured by replicate experiment. Notches represent 95% confidence intervals. Lower case letters indicate significant difference based on ls means with Tukey adjustment (P<0.05).

Table S1. Mean values for Fv/fm, chlorophyll, dry matter (DM), crude protein water soluble carbohydrates (WSC), neutral detergent fibre (NDF), acid detergent fibre (ADF) and acid detergent lignin (ADL) for all grasses in each condition

| Grass | Condition | Fv/fm | Chlorophyll µg/mL | DM % | Crude protein % | WSC  % | NDF % | ADF % | ADL % |
| --- | --- | --- | --- | --- | --- | --- | --- | --- | --- |
| Aber Zeus | CTRL20 | 0.80 | 16.38 | 17.26 | 24.25 | 3.26 | 34.72 | 11.56 | 2.80 |
| Aber Echo | CTRL20 | 0.82 | 7.36 | 11.64 | 23.12 | 4.24 | 31.59 | 11.92 | 2.86 |
| Aber Niche | CTRL20 | 0.80 | 8.25 | 14.06 | 21.19 | 5.57 | 29.42 | 11.38 | 2.31 |
| Aber Clyde | CTRL20 | 0.80 | 12.22 | 16.12 | 21.14 | 4.66 | 34.08 | 12.62 | 2.24 |
| Barolex | CTRL20 | 0.81 | 10.72 | 15.88 | 27.06 | 1.64 | 38.41 | 14.39 | 2.33 |
| Premium | CTRL20 | 0.81 | 12.06 | 15.88 | 23.21 | 3.62 | 36.92 | 14.00 | 2.66 |
| Aber Root | CTRL20 | 0.83 | 12.20 | 13.63 | 22.69 | 4.02 | 33.70 | 13.80 | 3.32 |
| Aber Dart | CTRL20 | 0.82 | 12.09 | 18.27 | 15.67 | 3.74 | 33.65 | 11.85 | 2.42 |
| Da vinci | CTRL20 | 0.80 | 12.67 | 18.40 | 23.73 | 4.71 | 34.47 | 11.24 | 1.33 |
| Aber Glyn | CTRL20 | 0.83 | 7.23 | 14.72 | 21.87 | 2.74 | 34.80 | 13.45 | 1.42 |
| Aber Zeus | CTRL50 | 0.79 | 9.02 | 12.57 | 14.71 | 1.61 | 33.80 | 13.51 | 2.41 |
| Aber Echo | CTRL50 | 0.83 | 8.32 | 10.87 | 17.12 | 1.97 | 34.50 | 14.64 | 4.11 |
| Aber Niche | CTRL50 | 0.82 | 7.60 | 12.65 | 21.15 | 4.89 | 35.04 | 14.24 | 3.73 |
| Aber Clyde | CTRL50 | 0.82 | 10.09 | 12.46 | 26.41 | 1.80 | 35.17 | 13.80 | 2.45 |
| Barolex | CTRL50 | 0.82 | 10.41 | 18.49 | 17.26 | 1.53 | 39.72 | 16.54 | 2.79 |
| Premium | CTRL50 | 0.81 | 10.37 | 14.43 | 15.48 | 1.55 | 41.43 | 17.09 | 2.23 |
| Aber Root | CTRL50 | 0.81 | 6.59 | 11.03 | 20.80 | 1.38 | 40.15 | 19.94 | 2.05 |
| Aber Dart | CTRL50 | 0.82 | 9.42 | 13.35 | 19.54 | 3.68 | 41.02 | 20.90 | 2.10 |
| Da vinci | CTRL50 | 0.80 | 10.44 | 13.42 | 14.32 | 2.06 | 41.39 | 18.61 | 1.49 |
| Aber Glyn | CTRL50 | 0.82 | 8.54 | 11.57 | 20.68 | 1.84 | 38.01 | 15.48 | 1.21 |
| Aber Zeus | Flood | 0.76 | 4.75 | 9.75 | 20.54 | 2.77 | 39.68 | 16.63 | 2.88 |
| Aber Echo | Flood | 0.81 | 3.68 | 10.38 | 20.06 | 3.43 | 45.84 | 21.79 | 3.52 |
| Aber Niche | Flood | 0.82 | 7.34 | 10.13 | 23.37 | 3.19 | 42.07 | 17.71 | 2.02 |
| Aber Clyde | Flood | 0.82 | 7.55 | 11.94 | 20.70 | 2.96 | 40.43 | 17.08 | 2.30 |
| Barolex | Flood | 0.79 | 9.04 | 10.37 | 21.80 | 0.64 | 42.73 | 20.05 | 3.09 |
| Premium | Flood | 0.78 | 3.00 | 8.91 | 21.54 | 1.73 | 43.11 | 19.10 | 2.39 |
| Aber Root | Flood | 0.82 | 4.52 | 9.07 | 21.58 | 1.75 | 43.74 | 19.33 | 2.70 |
| Aber Dart | Flood | 0.76 | 6.60 | 9.68 | 21.95 | 1.93 | 42.55 | 17.75 | 3.73 |
| Da vinci | Flood | 0.81 | 5.01 | 10.91 | 22.49 | 2.50 | 41.19 | 16.88 | 3.87 |
| Aber Glyn | Flood | 0.82 | 10.43 | 10.81 | 22.22 | 2.18 | 37.47 | 14.16 | 4.53 |
| Aber Zeus | Drought | 0.81 | 11.72 | 16.36 | 28.38 | 2.59 | 33.92 | 10.64 | 3.03 |
| Aber Echo | Drought | 0.74 | 11.76 | 19.53 | 26.44 | 4.33 | 33.82 | 13.37 | 0.96 |
| Aber Niche | Drought | 0.78 | 7.83 | 18.73 | 24.62 | 3.92 | 37.59 | 19.17 | 1.43 |
| Aber Clyde | Drought | 0.82 | 9.29 | 13.66 | 23.94 | 2.71 | 38.60 | 19.03 | 1.47 |
| Barolex | Drought | 0.65 | 12.97 | 17.25 | 29.27 | 2.44 | 35.27 | 12.95 | 0.75 |
| Premium | Drought | 0.53 | 11.50 | 26.32 | 27.30 | 3.77 | 33.44 | 10.41 | 1.00 |
| Aber Root | Drought | 0.81 | 5.84 | 11.78 | 26.16 | 2.56 | 32.83 | 13.17 | 1.19 |
| Aber Dart | Drought | 0.79 | 8.95 | 21.10 | 28.23 | 3.82 | 33.50 | 13.87 | 0.98 |
| Da vinci | Drought | 0.79 | 14.92 | 26.82 | 24.96 | 4.63 | 37.66 | 16.22 | 1.12 |
| Aber Glyn | Drought | 0.82 | 5.76 | 11.85 | 27.66 | 3.27 | 38.10 | 18.28 | 1.52 |
| Aber Zeus | HS | 0.77 | 8.45 | 18.04 | 25.89 | 1.54 | 32.66 | 13.07 | 1.66 |
| Aber Echo | HS | 0.76 | 5.68 | 15.01 | 20.79 | 4.14 | 31.27 | 11.50 | 1.12 |
| Aber Niche | HS | 0.77 | 6.82 | 14.25 | 24.14 | 3.09 | 34.01 | 14.63 | 1.26 |
| Aber Clyde | HS | 0.78 | 7.31 | 15.45 | 21.30 | 4.88 | 36.28 | 16.43 | 1.92 |
| Barolex | HS | 0.73 | 8.01 | 17.75 | 28.55 | 1.42 | 35.02 | 14.51 | 2.44 |
| Premium | HS | 0.77 | 10.20 | 14.51 | 27.96 | 4.31 | 32.98 | 12.60 | 2.23 |
| Aber Root | HS | 0.76 | 6.27 | 15.46 | 25.87 | 2.64 | 32.91 | 14.44 | 0.89 |
| Aber Dart | HS | 0.78 | 10.18 | 13.11 | 26.77 | 1.77 | 33.37 | 13.99 | 1.29 |
| Da vinci | HS | 0.73 | 11.19 | 19.60 | 24.27 | 3.54 | 36.87 | 16.42 | 3.30 |
| Aber Glyn | HS | 0.77 | 4.33 | 13.30 | 24.23 | 2.99 | 37.50 | 17.40 | 3.21 |

Table S2. Full compound list of VOC’s determined. (EXCEL FILE).

Table S3. Pairwise comparisons (manylm) of VOC profiles from Festulolium grown under different growth conditions, then incubated in rumen fluid for 24 h. Denominator degrees of freedom = 1, residual degrees of freedom = 47, LR = likelihood ratio. Significant results are given in bold.

|  | Control 2050 | | Drought | | Flood | | Heat shock | |
| --- | --- | --- | --- | --- | --- | --- | --- | --- |
|  | LR | *P*(adj) | LR | *P*(adj) | LR | *P*(adj) | LR | *P*(adj) |
| Control 2020 | **717** | **0.040** | **2080** | **0.020** | **2030** | **0.020** | **2120** | **0.020** |
| Control 2050 |  |  | **1270** | **0.020** | **1350** | **0.020** | **1450** | **0.020** |
| Drought |  |  |  |  | **664** | **0.020** | **564** | **0.050** |
| Flood |  |  |  |  |  |  | **936** | **0.020** |

Table S4. Classification success of random forests in assigning VOC profiles to the correct group.

| Group | Number correct | Error rate | Error rate (reduced compound panel) |
| --- | --- | --- | --- |
| AberClyde | 0/15 | 1.00 | NA |
| AberDart | 0/15 | 1.00 | NA |
| AberEcho | 1/15 | 0.93 | NA |
| AberGlyn | 2/15 | 0.87 | NA |
| AberNiche | 0/15 | 1.00 | NA |
| AberZeus | 2/15 | 0.87 | NA |
| Barolex | 3/15 | 0.80 | NA |
| Aber Root | 0/15 | 1.00 | NA |
| DaVinci | 0/15 | 1.00 | NA |
| Premium | 0/15 | 1.00 | NA |
| Control 2020 | 26/30 | 0.13 | 0.13 |
| Control 2050 | 23/30 | 0.23 | 0.23 |
| Drought | 21/30 | 0.30 | 0.23 |
| Flood | 24/30 | 0.20 | 0.13 |
| Heat shock | 24/30 | 0.20 | 0.17 |
| Experiment 1 | 49/50 | 0.02 | 0.02 |
| Experiment 2 | 50/50 | 0.00 | 0.00 |
| Experiment 3 | 50/50 | 0.00 | 0.00 |
